# Pilot and Feasibility Study of an Individualized Telehealth Exercise Program for People with Cystic Fibrosis

**DOI:** 10.3390/jfmk11020136

**Published:** 2026-03-26

**Authors:** Jordan Saag, Jonathan Bergeron, Julianna Bailey, Kathryn Monroe, Heather Hathorne, George M. Solomon, John D. Lowman, Surya P. Bhatt, Bryan Garcia, Stefanie Krick

**Affiliations:** 1 Department of Medicine, The University of Alabama at Birmingham, Birmingham, AL 35294, USA; jordanlsaag@uabmc.edu; 2 Gregory Fleming James Cystic Fibrosis Research Center, The University of Alabama at Birmingham, Birmingham, AL 35294, USAjlowman@uab.edu (J.D.L.);; 3 Division of Pulmonary and Critical Care Medicine, Department of Medicine, The University of Alabama at Birmingham, Birmingham, AL 35294, USA; 4 Department of Physical Therapy, School of Health Professions, The University of Alabama at Birmingham, Birmingham, AL 35294, USA

**Keywords:** cystic fibrosis, exercise, telehealth, acute and chronic airway diseases, prevention

## Abstract

**Background**: The Cystic Fibrosis Foundation (CFF) recognizes exercise as a critical part of managing cystic fibrosis (CF). This becomes even more important in the era of highly effective modulator therapy (HEMT) due to many people with cystic fibrosis (pwCF) having decreased symptom burden and a newfound ability to tolerate exercise better. Our single-center pilot study was designed to assess the implementation of a remotely delivered, individualized, and comprehensive exercise program for pwCF. We aimed to determine the feasibility, safety and acceptance of this intervention. **Methods**: PwCF ≥ 18 years old were recruited and consented at the University of Alabama in Birmingham in 2022 and 2023. Basic fitness was assessed for each participant, and an individualized exercise prescription was prepared for each participant, who was expected to exercise three times weekly on a remote basis with the exercise physiologist for 12 consecutive weeks. Subjects were reassessed at 4 and 7 months for post-exercise evaluation. Patient demographics and clinical parameters, including exacerbation rate, FEV_1_ percent predicted, 6-min walk test (6MWT), and modified shuttle test (MST) were collected. Questionnaire data from the CFQ-R, PRAISE, and IPAQ were also recorded. The study was registered with ClinicalTrials.gov (NCT04680403) and was submitted on 17 December 2020. **Results**: Our goal was to enroll 12 participants over the 2-year study period. We were able to recruit nine people for the study, with four participants finishing the program. From the 36 sessions offered over the 12-week program, participants completed an average of 15 sessions. Clinical outcome data was observed, including lung function and exacerbation frequency, but not statistically analyzed due to the small sample size. **Conclusions**: Implementation of an individualized telehealth-based exercise program for pwCF was well received by participants, safe, and appreciated by the participants. Recruitment and adherence were challenging, which was partially due to the ongoing pandemic. Follow-up studies are needed to assess whether improvements in reducing the amount or supervision of weekly exercise sessions and/or extending the total time might help with adherence.

## 1. Introduction

Exercise is recommended in consensus documents for the care management of people with cystic fibrosis (pwCF) and represents an important part of the comprehensive treatment to improve quality and quantity of life [1,2]. Exercise may be even more beneficial in an era of cystic fibrosis transmembrane conductance regulator (CFTR) modulator therapies, as exercise has been suggested to exert anti-inflammatory effects, thereby potentially augmenting residual CFTR activity [3,4]. The CF Foundation promotes regular physical activity by suggesting that exercise improves lung function and bone health [2,5]. Airway clearance techniques and exercise have been historic mainstays of treatment for pwCF. However, due to the time commitment for the many medical treatments that pwCF already have, adherence to exercise and even their regular treatments has been problematic in the past and are not well studied [6,7]. Radtke et al. discussed in a meta-analysis including over 20 randomized controlled trials with over 800 participants that physical activity interventions, including aerobic and strength exercises for six months or longer, led to a potential improvement in exercise capacity without a significant benefit for lung function or quality of life, but most of those studies were done in children with CF and were conducted before the introduction of highly effective modulator therapies (HEMT) [8].

The era of HEMT has changed this paradigm to some degree, whereby the treatment burden from regular airway clearance may require a more individualized approach, while consistent exercise remains a recommendation [9,10]. Taking together previous reports and guidelines, exercise should be an integral part of CF care, and while there is a high degree of interest in the topic, standardized exercise testing and training programs appear to be underused throughout the United States and the rest of the world [11,12].

The CF Foundation (CFF) stresses the importance of exercise as a daily routine [2]. PwCF should work with their CF care teams to develop an individualized exercise program, but not all CF care teams have the resources to include a specialized physical therapist [13]. Most general physical therapists or fitness coaches are not trained to take into consideration CF-related issues and comorbidities when developing individualized fitness programs. If we achieved an 80% attendance rate to our exercise prescription, we would anticipate a 2–3-fold improvement in dyspnea scores and quality of life in our participants with CF, as reported in several trials studying the role of exercise on CF-related health outcomes [14,15,16].

We agree with the phrase, “Exercise is Medicine in Cystic Fibrosis,” but we need to identify the best method of delivering and enhancing adherence to this “medicine” and ensuring access for all pwCF. This study was developed to help determine the feasibility of a novel and accessible model of exercise prescription delivery for cystic fibrosis to enhance adherence, and ultimately improve quality of life with CF, particularly in the post-modulator therapy era.

We hypothesized that a combination of a comprehensive video–telehealth exercise prescription for people with CF is accessible, feasible, and can improve adherence to an exercise regimen, thereby improving health outcomes. Our study included the assessment of administrative feasibility and adherence.

## 2. Materials and Methods

### 2.1. Study Population

This small single-center prospective pilot study was conducted at the University of Alabama, Birmingham, Adult Cystic Fibrosis Center. People with a diagnosis of CF who were greater than 18 years of age were recruited and consented between 2022 and 2023; patients with a CF exacerbation within the preceding 6 weeks and patients who did not want to or could not exercise were excluded. The study was conducted in accordance with the principles outlined in the Declaration of Helsinki and approval was obtained from the UAB Institutional Review Board on 17 February 2021 (No. 300006495). The study was registered with ClinicalTrials.gov (ID NCT04680403) and was submitted on 17 December 2020.

### 2.2. Exercise Delivery and Prescription

The subjects were initiated on telehealth exercises at home via a data-enabled smartphone with video capabilities facilitating live and interactive two-way video-conferencing using a HIPAA (Health Insurance Portability and Accountability Act)-compliant app (Avizia by American Well, Boston, MA, USA). Participants received a heart rate monitor, pedometer, exercise mat, resistance exercise bands and an exercise training manual. In addition, to support adherence to the exercise prescription, a daily logbook was provided for participants to complete. The comprehensive exercise prescription was based on position papers from the American College of Sports Medicine as well as the European CF Society’s Exercise Working Group, and included both aerobic and strength/power training.

Aerobic training: Overground or treadmill walking/jogging, stair climbing, or cycling; 40–60% of HR reserve and/or perceived exertion or dyspnea 3–5/10 (Borg CR10; moderate to severe) for 20–30 min/day, 3–5 days/wk. (1) Duration may start at 2x 10 min sessions, with the goal of a 1x 30 min session by the end of training. (2) Resting HR was reassessed biweekly and training HR range adjusted accordingly.

Strength/power training: Resistance bands and body weight/calisthenics, (based on participant preference and ability). 2 days/wk focused on “traditional” resistance training (e.g., knee extension, bicep curls, shoulder rows); 1 day/wk involved power training (e.g., squat jumps, lunges, sprints); 70–80% of 1RM (~12 to 8 rep max); 2–4 sets of 8–12 repetitions of key muscle groups for 3 days/wk (48 h between bouts). (1) When participant could complete two complete sets of 12 repetitions (with good form), a third set was added, (2) when the third set of 12 repetitions could be completed, then the intensity/weight was increased to the next increment.

Vital signs were reported by the subjects and not remotely monitored during the training. In general, the prescription aimed to achieve moderate-level exercise sustained throughout the session and was subjectively confirmed by periodically obtaining a rate of perceived exertion (RPE) score.

A trained exercise physiologist scheduled the sessions individually with each participant and was guiding the telehealth sessions by watching the participant exercising via a telehealth interface, and advised how to do the exercises properly while correcting positions and forms if needed. Every exercise session for each participant was individualized depending on what equipment was present in their homes in addition to the provided equipment. Each participant continued with their prescribed exercises throughout the course of the sessions and only increased repetitions when they felt stronger and per suggestion by the exercise physiologist.

Exercise devices such as treadmills or bikes were not under monitoring or control by our researchers. Safety criteria that were applied by the exercise physiologist during each exercise session included the monitoring of the participant’s heart rate, subjective effort, as well as periodic verbal check-ins to monitor signs of unproportional stress such as dyspnea, weakness, dizziness, pain, and specifically chest pain or tightness. Moderate exercise was not always achievable and depended on various factors including worsening respiratory symptoms, generalized fatigue, and need for recovery. Breathing exercises chosen with patient preference were also incorporated. Options included diaphragm and pursed lips breathing or breath retention training. Sessions then ended with a quick cool-down stretch. There were no limitations on exercising outside of supervised sessions if desired.

### 2.3. Outcome Measures

All participants attended a baseline visit where demographic information was collected, and basic fitness was assessed by an exercise physiologist who prepared an individualized exercise prescription.

Primary outcomes were feasibility, safety, and adherence. The assessment of feasibility included participant recruitment and retention and our site infrastructure readiness. Safety criteria were described above and adherence was assessed by interrogation of the participants’ log books regarding the number of sessions attended compared to number of planned sessions and their presence at follow-up appointments.

Participants were then followed up at 4 and 7 months for post-exercise assessments. Participant demographics and clinical parameters, including absolute and percentage change in forced expiratory volume in one second (FEV_1_), percent predicted (ppFEV_1_), 6MWT distance, MST distance, CFQ-R, IPAQ, and PRAISE questionnaires were obtained at all three visits [17,18,19]. Exacerbation frequency was also measured in the year before and after the intervention. Exacerbations were defined as the sudden onset of respiratory symptoms including congestion and productive cough with or without a decrease in lung function requiring oral or intravenous antibiotics in the in- or outpatient setting [20,21]. The study protocol was approved by the Human Studies Subcommittee of the Institutional Review Board of the University of Alabama at Birmingham on 17 February 2021 (IRB-300006495).

### 2.4. Statistical Analysis

The primary outcomes of the study were feasibility, safety, and adherence to the study protocol. Secondary outcomes included the aforementioned clinical parameters, change in ppFEV_1_, exacerbation frequency, 6MWDT distance, MST distance, and questionnaire data. The limited sample size did not allow for meaningful inferential statistical analysis. Data was graphed for descriptive intent. Only participants who completed testing at all three time points were included in the analysis of that particular clinical outcome.

## 3. Results

### 3.1. Recruitment and Retention

**Figure 1:**
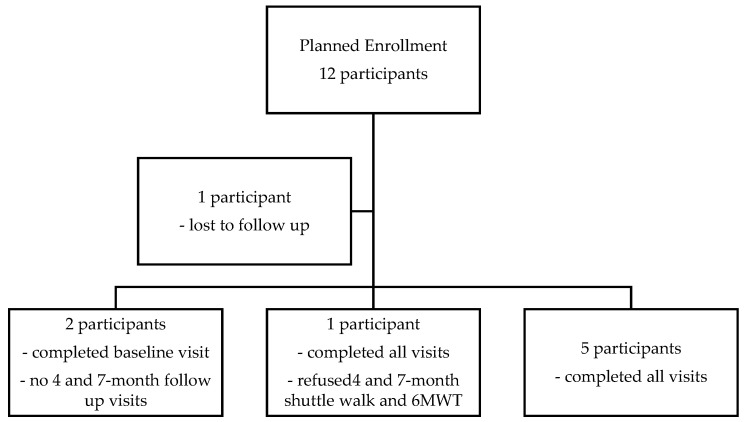
Flowchart.

We enrolled nine participants over a 2-year study period. Four participants were male and seven were Caucasian, one Hispanic, and one African American, which is representative of the CF population in our region. The initial goal was set to 12 participants, and this proved to be significantly impacted by the logistics and socioeconomic complications of the COVID-19 pandemic. Detailed information about recruitment and retention is found in the flow chart and the table showing baseline characteristics of the participants who enrolled and started the study (Figure 1 and Table 1), which includes baseline characteristics, including FEV1% before enrollment and the number of exacerbations 12 months before enrollment. We also included whether participants were taking highly effective modulator therapy (ETI—elexacaftor/texacaftor/ivacaftor).

There were a total of 36 telehealth exercise sessions offered over 12 weeks, with the remaining six participants completing an average of 15 sessions (range of 3–27). Reasons for missed sessions included fatigue preventing the participant from scheduling the exercise session, CF exacerbation (subject was on either intravenous or oral antibiotics and did not feel well enough to exercise), hospitalization, arthritis flare, or holiday plans (subject was traveling and not able to connect remotely for the sessions). Detailed information pertaining to the exercise regimen for each participant can be seen in Table 2. Regarding the follow-up research visits, there was also variable attendance and completion of the clinical outcomes at baseline, 4 and 7 months. Of the remaining six participants, all completed the required FEV_1_ testing, five completed the IPAQ, and only four completed CFQ-R, PRAISE, 6MWT, and MST. No adverse events related to the exercise sessions occurred over the study period.

### 3.2. Effect on Lung Function

**Figure 2:**
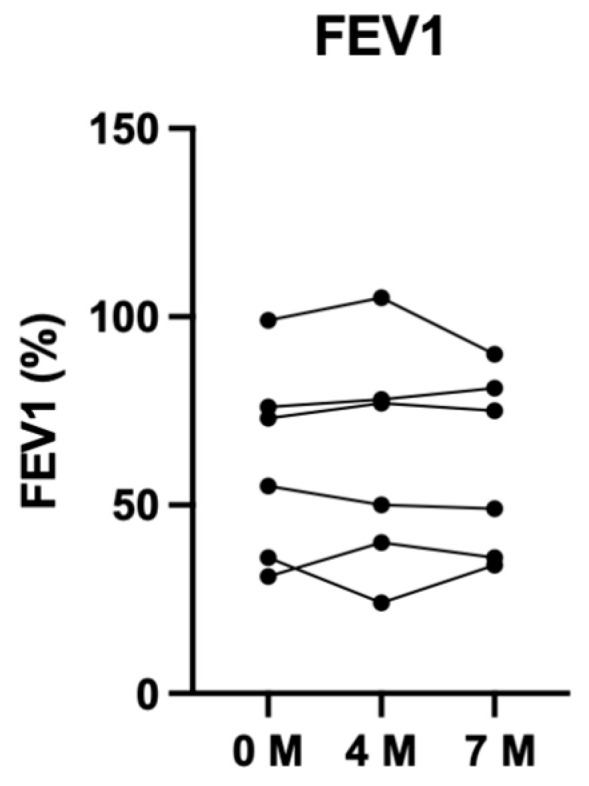
Change in ppFEV_1_ per participant over 7 months.

Absolute and percentage change in ppFEV_1_ were measured over the three time points (baseline, 4 and 7 months). Among the six participants who completed testing, there was no discernable change in lung function following the intervention. The baseline mean ppFEV_1_ was 62%, the 4-month mean was 62%, and the 7-month mean was 61%. This equated to a −1.3 absolute difference (−2.1%) from baseline to 7 months. Visual depiction of this data is displayed in Figure 2.

### 3.3. Exercise Effect on CF Exacerbation Rate

**Figure 3:**
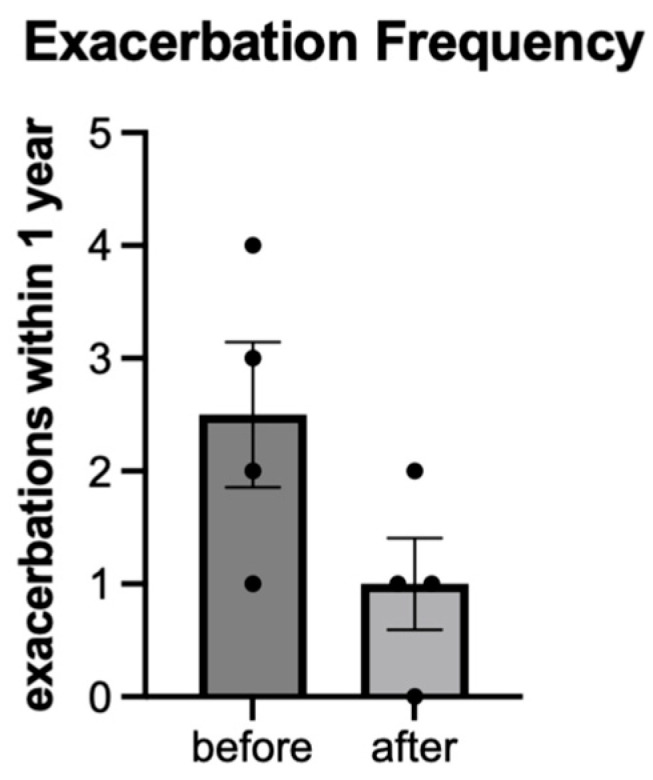
Exacerbation frequency in the year before and after exercise intervention.

The CF exacerbation rate was measured during a one-year period before and after the intervention. This data was compiled from the four participants who completed testing for all other clinical outcomes. The average number of exacerbations decreased from 2.5 (1.3 SD) to 1 (0.8 SD) (Figure 3). This amounts to a 60% observed trend decline in exacerbations in a small subgroup and we did not infer significance given the small sample size.

### 3.4. Effect of the Intervention on Other Secondary Outcomes

**Figure 4:**
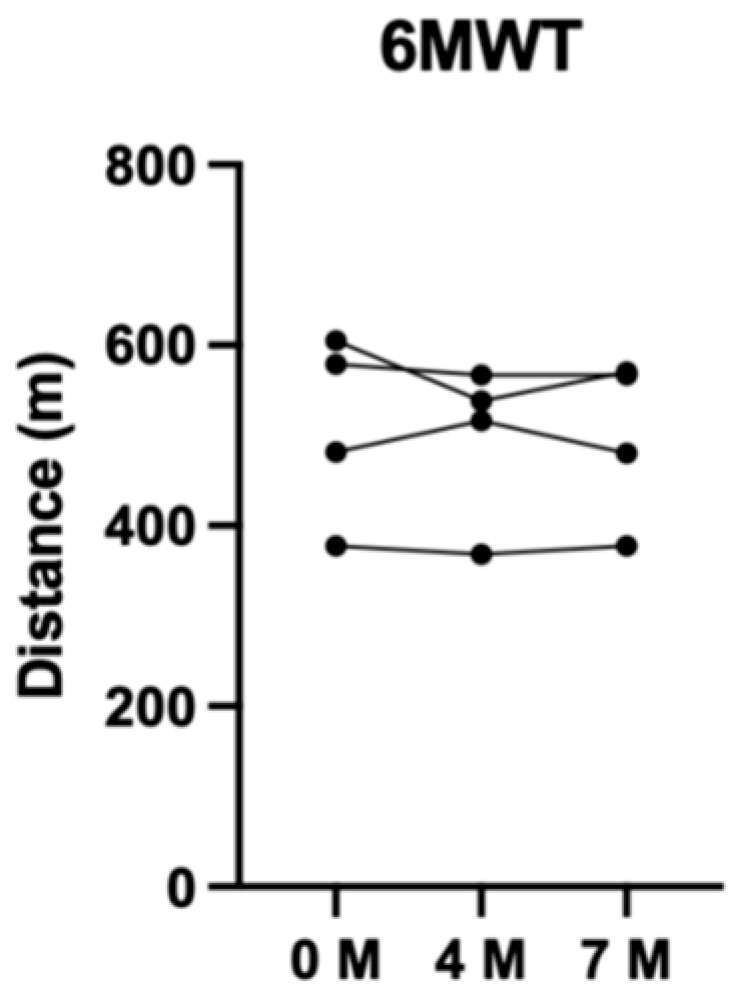
Distance completed during 6MWT over 7 months.

**Figure 5:**
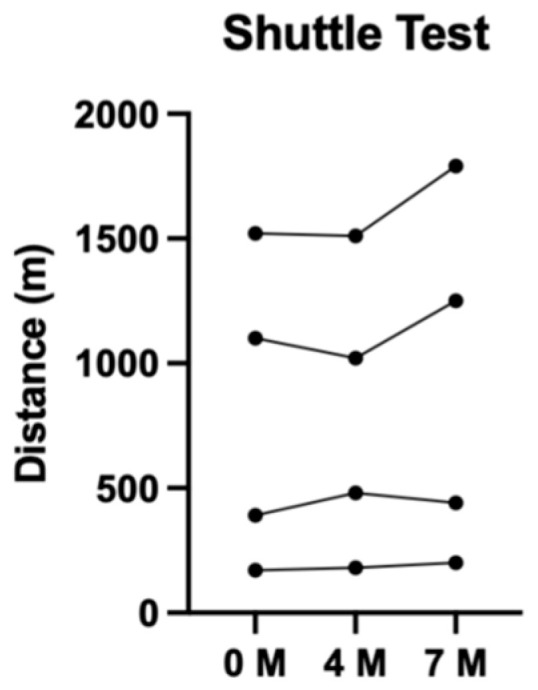
Distance completed during modified shuttle test over 7 months.

Measures of physical fitness, including the 6MWT and MST, were recorded over the three time points (Figure 4 and Figure 5). Notably, there was no clear difference in the mean change in 6MWT distance over 7 months, measuring to a −12 m (−2%) change from baseline. The MST revealed more positive results with a mean change in distance of 125 m (+15%) over 7 months.

**Figure 6:**
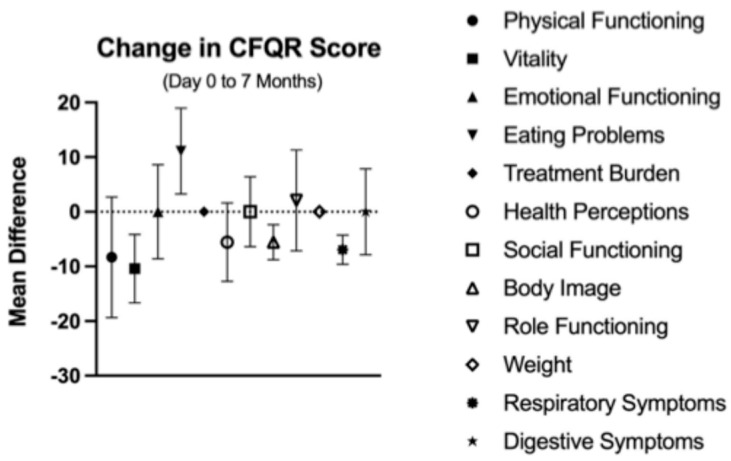
Mean change in CFQ-R categorical scores over 7 months.

**Figure 7:**
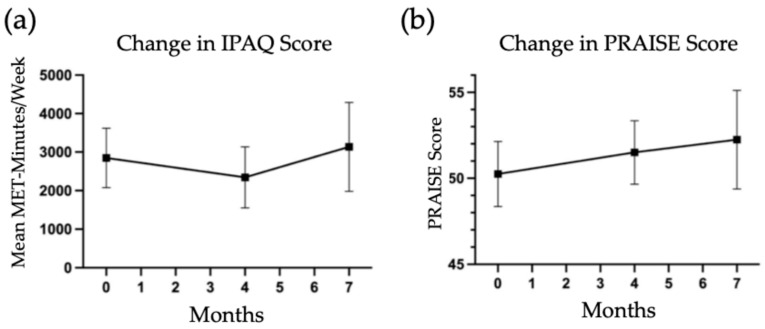
(**a**) Mean MET-minutes/week over 7 months; (**b**) mean PRAISE score over 7 months.

Questionnaire data from the CFQ-R, IPAQ, and PRAISE surveys were collated among the participants who completed measures at all three time points. CFQ-R scores separated by category are graphed in Figure 6, showing mean changes from baseline to 7 months. The categories with standard deviations outside of zero include a positive change in eating problems, but negative change in vitality, body image, and respiratory symptoms. IPAQ score is demonstrated in Figure 7a, with mean MET-minutes per week graphed over 7 months. The absolute difference in metabolic equivalent (MET)-minutes per week was 287.5, a change of 10.1% from baseline. Mean PRAISE score (seen in Figure 7b) also showed a modest increase over 7 months, with an absolute increase of 2 (4%) from baseline. Both error ranges for IPAQ and PRAISE values were quite large, attributable to the small sample size.

## 4. Discussion

Our pilot study of a model for an individualized, telehealth-based exercise prescription for pwCF was safely implemented and addressed both administrative and scientific feasibility, but clearly showed a lack of feasibility in a very selective cohort with a high time commitment expected from the participant. However, this is comparable to previous exercise studies in people with cystic fibrosis, whereby comparable studies have similar, though slightly higher, target recruitment numbers [8]. From a process standpoint, the study was completed on time and within budget. Recruitment was challenging; however, there was good engagement and feedback from the participants who were not lost to follow up.

Addressing the scientific feasibility, adherence, and fallout from the program proved to be a major obstacle. However, we feel that the specific clinical outcomes reflect a good variety of both objective and subjective measures of pulmonary function and fitness. Furthermore, they were safe and easy to obtain during the three individual visits. The limited sample size prohibits inferential statistics and assumptions on significance, but there are still interesting observations to draw from the data. First and foremost was the 60% reduction in exacerbation frequency observed, which could also have been affected by the ongoing pandemic leading to social distancing and routine masking, which altered the epidemiology of viral and bacterial transmission. This large of an effect was also not seen with other objective measures, as ppFEV_1_ and 6MWT distance had little to no change, and MST distance had only modest improvement. The survey data, including CFQ-R categories, IPAQ, and PRAISE, did not reveal any noticeable trends. Therefore, it is unclear if the large reduction in exacerbations will hold with a larger sample size or eventually correlate with the other measures of pulmonary function and physical conditioning. Assuming a similar effect size of 60%, a calculated sample size of 22 participants would be necessary to achieve statistical significance with a *p*-value of 0.05 and power of 0.8. Since the sample size was very small and participants were heterogenous in demographics, baseline health, and reported exacerbations during the study period, the questionnaire data also has to be interpreted cautiously and will require a more robust sample size.

As stated previously, recruitment and adherence were the primary detractors from our pilot study. We also recruited from a relatively unique cohort of older patients with worse lung function at baseline compared to those seen in the PROMISE study [22]. In comparison, the ACTIVATE-CF trial was a non-telehealth study but had similar problems with recruitment and attrition, only achieving 40% of their target sample size with a 56% adherence rate to the intervention [23]. Telehealth-based exercise programs have been successfully implemented with larger sample sizes, albeit in different populations. For example, one study evaluated a virtual exercise program for people with knee osteoarthritis and had 90% adherence over a 12-month study period [24]. Differences could be due to a larger recruitment population and a less intense exercise program. In addition, people with CF have a higher burden of care, already spending several hours per day on therapy when compared to most other patients with chronic diseases [2,5,10,25]. Another study investigating a telerehabilitation program for people with chronic obstructive pulmonary disease (COPD) demonstrated 96% adherence overall, and a 100% adherence in the telerehabilitation group alone [26], which again could be due to a higher prevalence of COPD, and many people with COPD being older or on disability and not having conflicting work schedules. Telehealth-based exercise interventions have proven to be feasible on a large scale, and we believe our protocol can have similar applications. Potential strategies to improve adherence for our intervention include reducing the number of total sessions but increasing the time per session, or incorporating a self-guided video option instead of a monitored virtual visit. Considering the clinical outcome parameters, our results are reassuring in that, despite a small sample size, lung function remained stable in the participants over the time the exercise was delivered up until 7 months follow up. Harris et al. assessed spirometry right after the delivery of a single bout of maximal exercise in 33 participants, leading to an acute improvement in pulmonary lung function, but it is not clear whether this would be sustainable. Most previous studies were done pre-CFTR-modulator era and cohort sizes were 31–65 participants, including trials lasting over a year with unsupervised exercise training. Most of these studies showed either an improvement in lung function or a delay in decline, but exercise prescriptions were very different, ranging from submaximal exercise mirroring daily living activities to aerobic exercise targeting a heart rate of 150 beats/min and observations over 3 years [27,28,29]. A more recent meta-analysis including 24 randomized controlled trials (875 participants) had heterogenous results, implying that physical activity interventions for six months or longer led to a potential improvement in exercise capacity without a significant benefit for lung function or quality of life [8]. Most of the included studies assessed these outcomes in the pre-modulator era and only two studies included adult participants [29,30]. Therefore, future studies are needed—particularly multi-center trials—to establish the feasibility of determining optimal exercise prescriptions for individuals with cystic fibrosis receiving CFTR modulator therapy. Importantly, variability in exercise capacity should be considered, as patients who initiated modulator therapy earlier may demonstrate higher levels of physical fitness. We initially progressed participants to 8–12 repetitions, a range commonly used in exercise prescriptions. Current Cystic Fibrosis Foundation recommendations emphasize incorporating both functional and strength-based exercises as part of a comprehensive regimen, although supporting data remains limited. Therefore, future studies should further individualize exercise programs by accounting for baseline fitness, overall health status, and exercise variation to enhance adherence and improve exercise capacity. The aging cystic fibrosis population may be of particular interest, as postmenopausal changes in women and age-related changes in both men and women—including increased risk of osteoporosis—should be considered in program development. Alternative strengthening approaches, such as yoga or Pilates, may also offer benefits.

### Study Limitations

As mentioned above, our study had several limitations with recruitment and adherence failure being two of the most obvious limitations. The study was conducted during the pandemic, which made it not only challenging to recruit patients but also to work with staff turnover to help recruit and deliver the exercise prescription. Delivery of an exercise regimen three times weekly was perceived too often for our patients and since these sessions were supervised, our exercise physiologist offered sessions within the working hours between 8 a.m. and 5 p.m., which was challenging for pwCF who worked full-time or had child care responsibilities. For the future, it might be beneficial to alternate supervised and unsupervised exercise sessions, which might lead to higher recruitment and adherence rates. Some patients might prefer solely unsupervised sessions with follow up phone calls for debriefings to see what worked for them and what did not. The exercises themselves were perceived safe and appreciated by the participants and they enjoyed being able to choose their regimen according to existing equipment at home. Depending on the baseline fitness, repetitions could be increased and different exercise modalities could be included, such as breathing exercises and balance exercises.

## 5. Conclusions

As multidisciplinary care for CF continues to evolve in the post-HEMT era, there will be a crucial need for exercise-based interventions to augment pharmacotherapy, help improve lung function, and protect from comorbid chronic health conditions such as cardiovascular disease or obesity-associated diseases, which will be on a rise as people with CF are getting older [31,32,33]. Although underpowered, this pilot study established our model as a safe exercise program that can be delivered in a telehealth format. It was well received by the participants who participated and could serve as a model to help meet recommended exercise goals. Nevertheless, due to a low adherence rate, our study did not demonstrate feasibility, especially if we were to try to deliver it to a broader CF population. Future endeavors to expand our work to a larger sample size will need the adjustment of our current protocol, potentially switching to less monitored and more self-guided sessions, with flexibility in the timing of the sessions.

## Figures and Tables

**Table 1:** Demographic and clinical characteristics of study participants.

Participant	Age	Ethnicity	Sex	BMI	FEV1%	HEMT	Exacerbations
1	33	Caucasian	male	21.79	55	ETI	4
2	57	Caucasian	female	44.31	73	ETI	0
3	33	Caucasian	female	20.01	76	ETI	2
4	37	Caucasian	female	28.92	31	ETI	3
5	35	Caucasian	male	39.71	99	ETI	1
6	51	Caucasian	female	21.92	36	ETI	3
7	26	Hispanic	male	21.43	50	none	3
8	26	African American	female	18.07	39	ETI	3

**Table 2:** Mean distribution (%) of types of exercises (aerobic, resistance or blended (exercises containing both resistance and aerobic components)) for each participant.

Participant	No. of Sessions	% Aerobic	% Resistance	% Blended
1	27	33.3	44.4	22.2
2	5	20	40	40
3	17	0	70.6	29.4
4	3	33.3	66.6	0
5	20	5	25	70
6	17	0	0	100

## Data Availability

The original contributions presented in this study are included in the article. Further inquiries can be directed to the corresponding author.

## Abbreviations

The following abbreviations are used in this manuscript:

CF	Cystic Fibrosis
HEMT	Highly effective modulator therapy
pwCF	People with Cystic Fibrosis
FEV_1_	Forced expiratory volume in 1 s
6MWT	6-min walk test
CFTR	Cystic Fibrosis transmembrane conductance regulator
CFQ-R	Cystic Fibrosis Questionnaire-Revised
IPAQ	International Physical Activity Questionnaire
PRAISE	Pulmonary Rehabilitation Adapted Index of Self-Efficacy
ppFEV_1_	Percent predicted forced expiratory volume in 1 s
SD	Standard deviation
MET	Metabolic equivalent
COPD	Chronic obstructive pulmonary disease
